# INR reduction after prothrombin complex concentrate (Co-fact^©^) administration: comparison of INR outcomes in different patient categories at the emergency department

**DOI:** 10.1186/1865-1380-6-14

**Published:** 2013-05-10

**Authors:** Floris Roodheuvel, Jack JM Ligtenberg, Jan C ter Maaten

**Affiliations:** 1 University Medical Center Groningen (UMCG), Groningen, The Netherlands

## Abstract

**Background:**

Co-fact^©^, prothrombin complex concentrate, is used for restoring the international normalized ratio (INR) in patients on vitamin K antagonists (VKA) presenting with acute bleeding. In this prospective cohort study, we evaluated whether adequate INR values were reached in ED patients using the Sanquin (Federation of Dutch Thrombosis Services) treatment protocol.

**Methods:**

We evaluated this protocol for two target INR groups: group 1, target INR ≤ 1.5 (for life-threatening bleeding/immediate intervention); group 2, target INR 1.6-2.1 (in cases of a minor urgent surgery or serious overdosing of anticoagulant). We specifically wanted to identify both under- and over-treated patients. Reversing VKA anticoagulation therapy to unnecessarily low INR values may involve thrombotic risks. Apart from this risk, the patient is also administered an excess amount of the drug. This means unnecessary costs and may present problems with restoring an anticoagulated state at a later time.

**Results:**

In our cohort, the Sanquin dosing protocol was followed for 45/60 patients. It appeared that out of the 41 patients in group 1 (target INR ≤ 1.5), 35 (85%) achieved the goal INR. This occurred more often than for the 19 patients in group 2 (target INR 1.6–2.1), where only 6 (32%) achieved the goal INR. Using the protocol resulted in a positive trend toward better INR reversal in group 1.

In group 2, no relation between using the protocol and achieving the desired INR value was detected. Physicians ignoring the proposed dose of Co-fact^©^ prescribed significantly less Co-fact^©^ (even when correcting for patient weight). It appeared that patients in group 1 had a significantly lower baseline INR than patients in group 2. Group 2 patients, on the other hand, had a baseline INR > 7.5 in 53% of the cases.

**Conclusion:**

In our cohort, for most patients in INR group 2 treated with Co-fact^©^, the achieved INR value was outside the desired range of 1.6-2.1. The supra-therapeutic range of baseline INR in group 2 may have contributed to the different kind of bleeding witnessed in this patient group.

Our results support the idea that treatment of patients on vitamin K antagonists with Co-fact^©^ could benefit from a slightly different approach, taking into account the INR value to which the patient needs to be reversed.

## Background

Many patients admitted to the emergency department (ED) use anticoagulants, mostly vitamin K antagonists (VKA). Newman et al. [1] showed that the majority of patients in the ED using VKA had a sub- or supra-therapeutic international normalized ratio (INR). Chronic anticoagulation is associated with an increased risk of bleeding, which is related to the degree of anticoagulation, e.g., the height of the INR [2]. Patients using VKA presenting to the ED with hemorrhage have increased morbidity and mortality compared with patients not using VKA.

In addition to resuscitation measures, standard treatment of VKA-associated bleeding includes improvement of coagulation status. Vitamin K, fresh frozen plasma, or prothrombin complex concentrate (PCC, Co-fact^©^) are the most used treatment options in the Netherlands.

Sanquin, the Federation of Dutch Thrombosis Services and manufacturer/supplier of Co-fact^©^, provides a dosing regimen (Additional file 1) in which the proposed amount of PCC to be administered is related to the INR at presentation, the weight of the patient, and the desired target INR. This desired target INR depends on the indication: a target INR ≤ 2.1 is the aim in case of a minor (urgent) surgery or serious overdosing of anticoagulant with bleeding complications, while a target INR ≤ 1.5 is the aim for severe bleeding complications with hemodynamic instability or cerebral bleeding, or if acute surgical intervention is needed [3,4].

Through regular use in our ED, we observed that treatment with Co-fact^©^ is not optimal in every case. Desired INR target values are not always reached, with patients being over-treated with PCC and thus reaching lower INR values than necessary. There are also patients in whom the INR stays above the desired value. Both these outcomes may be unfavorable for the patient. The risk of an INR value above the desired target in an actively bleeding patient is evident. On the other hand, reversing VKA anticoagulation therapy to unnecessarily low INR values may come with thrombotic risks. These risks may vary with each individual patient, depending on their indication for VKA therapy and risk stratum [5,6]. Apart from this risk, the patient is additionally administered an excess amount of drug that was not needed. This results in unnecessary costs and may present problems with restoring anti-coagulated state later on.

The aim of this study was to evaluate whether adequate INR values were reached when administering Co-fact^©^ to ED patients using the Sanquin treatment protocol. We evaluated the results of this protocol in two different INR target groups (group 1, target INR ≤ 1.5; group 2, target INR 1.6–2.1).

## Methods

### Patients

Sixty-two patients (female 20/male 42) admitted to our university hospital-based ED were included in a 13-month period from July 2010 to August 2011. Inclusion criteria were: the need for acute VKA reversal and age > 18 years. Exclusion criteria were administration of vitamin K, FFP, or PCC prior to the ED visit. There were no referrals from other medical institutions in our cohort. Two patients were excluded afterwards because of missing data such as endpoint INR values and desired target INR values.

### Study design

This was a prospective observational cohort study, in which regular patient care was provided. The decision to treat a patient with Co-fact^©^ was made by the treating physician; the investigator had no influence on this process. Approval from the ethics committee was waived since the study only observed treatment and no interventions were performed.

Physicians of included patients filled out an investigation form where patient characteristics were noted, such as age, sex, and estimated weight. The desired target INR, the admission INR, the amount of Co-fact^©^ administered, time between administering Co-fact^©^ and drawing a new blood sample for INR control, and the achieved INR value were noted. Following the protocol, Co-fact^©^ was administered at 2 ml/min. The control blood sample had to be drawn 15 min after the completed administration of the full dose.

The usage and amounts of vitamin K or fresh frozen plasma were noted. Eventually, physicians had to explain why they had not used the proposed dosing regimen.

In group 1 (target INR ≤ 1.5) inadequate VKA reversal was represented by values of INR > 1.5.

In analyzing the data for target group 2 (target INR 1.6–2.1), a deliberate difference was made between achieving an INR ≤ 2.1 or achieving an INR between 1.6 and 2.1. The reason for this was that we specifically wanted to identify both under- and over-treated patients. Over-treated patients could have been treated with less Co-fact^©^ than was originally administered, while under-dosed patients should have been treated with more. This is a different approach than the manufacturer of Co-fact^©^ uses. They state that any INR ≤ 2.1 is adequate for INR group 2 (target INR 1.6–2.1). When taking only the acute bleeding episode into account, this may be true. However, reversing INR to normal levels in a patient with no life-threatening bleeding may be unfavorable [6].

### Statistical analysis

Both baseline and endpoint data are presented as the mean ± standard deviation. Data are presented as case series with proportions and percentages when appropriate. Due to the small sample size, no significance testing was performed. Calculations, data storage, and statistical analysis were performed with Microsoft Excel, SPSS version 18, and GraphPad software.

## Results

The most relevant included patient categories were patients with intracranial bleeding, gastrointestinal bleeding, or requiring an acute intervention (diagnostic or treatment-related). There was no difference in age, weight, measured time, or use of vitamin K between INR target groups (Table 1). When vitamin K was administered, in 86% of the cases the dose was 10 mg. There was no difference in administration between the INR target groups and, as expected, no relation with achieving an adequate goal INR. When comparing estimated weight and measured weight, the mean difference was 2.48 kg (± 6.9 kg). Sanquin divides patients into 10-kg categories for dose calculation; the observed weight difference had no influence on dosing of our patients. Fresh frozen plasma (FFP) was administered only once; no further statistical analysis on this subject was performed.

**Table 1:** Cohort characteristics

	**Total cohort (*n *= 60)**	**Group 1 (*n *= 41)**	**Group 2 (*n *= 19)**
Male *N*	42/60 (70%)		
PCC Indication (n)			
Intracranial bleeding	21 (35%)	21	0
Gastrointestinal bleeding	14 (23.3%)	8	6
Pre-surgery/intervention	11 (18.3%)	6	5
Intramuscular hematoma	4 (6.7%)	0	4
Postoperative bleeding	3 (5.0%)	1	2
Post-traumatic bleeding	2 (3.3%)	2	0
Pulmonary bleeding	2 (3.3%)	1	1
Pharyngeal bleeding	1 (1.7%)	0	1
Hematuria (unstable patient)	1 (1.7%)	1	0
Bleeding with acute abdominal aneurysm	1 (1.7%)	1	0
Mean age (years) (SD)	69 (± 12.7)	70.1 (± 12.4)	66.8 (± 13.3)
Mean estimated weight	80.5 (± 13.7)	81.5 (± 12.6)	78.2 (± 15.9)
Baseline INR	5.23 (± 2.96)	4.20 (± 2.13)	7.44 (± 3.32)
Mean PCC dose (ml)			
Dosing table used	66.2 (± 22.1)	74.6 (± 22.5)	52.5 (± 12.5)
Dosing table not used	45.3 (± 19.6)	48.5 (± 19.1)	25 (± 7.1)
Combined	61.0 (± 23.2)	66.3 (± 24.6)	49.5 (± 14.7)

There was a great difference in both baseline INR values and in the amount of Co-fact^©^ administered between groups 1 and 2.

Of the 41 patients in group 1, the protocol was followed in 28, and 26 (93%) reached their target INR (Table 2). This was more often than for patients in group 2: of the 19 patients in this group, the protocol was followed in 17 patients, and only 5 patients (32%) reached their target INR. Out of the 13 patients in group 1 where the protocol was not followed, 9 (69%) reached their target INR. There were only two cases in group 2 where the protocol was not followed. Out of the 19 patients in group 2, 13 patients did not reach the target INR range: 9 patients had INR values ≤ 1.5 and 4 patients > 2.1.

**Table 2:** Results reaching target

	**INR ≤ 1.5**	**INR 1.6**-**2.1**
Target INR reached/total (%)	35/41 (85%)	6/19 (32%)
Dosing table used	28/41 (68%)	17/19 (89%)
Target reached when dosing table used	26/28 (93%)	5/17 (29%)
**Results dosing table**	**Dosing table**	**No dosing table**
INR ≤ 1.5 reached/total (%)	26/28 (93%)	9/13 (69%)
INR 1.6-2.1 reached/total (%)	5/17 (29%)	1/2 (50%)

## Conclusions and discussion

In our cohort, the Sanquin dosing protocol was followed for 45 patients and not followed for 15 patients. Patients in group 1 (target INR ≤ 1.5) achieved the goal INR significantly more often than patients in group 2 (Target INR 1.6–2.1), even when the protocol was ignored. Using the protocol resulted in a positive trend toward better INR reversal in group 1. In group 2, no relation between using the protocol and achieving the desired INR value was detected. Physicians ignoring the proposed dose of Co-fact^©^ prescribed significantly less Co-fact^©^ (even when correcting for patient weight).

**Figure 1:**
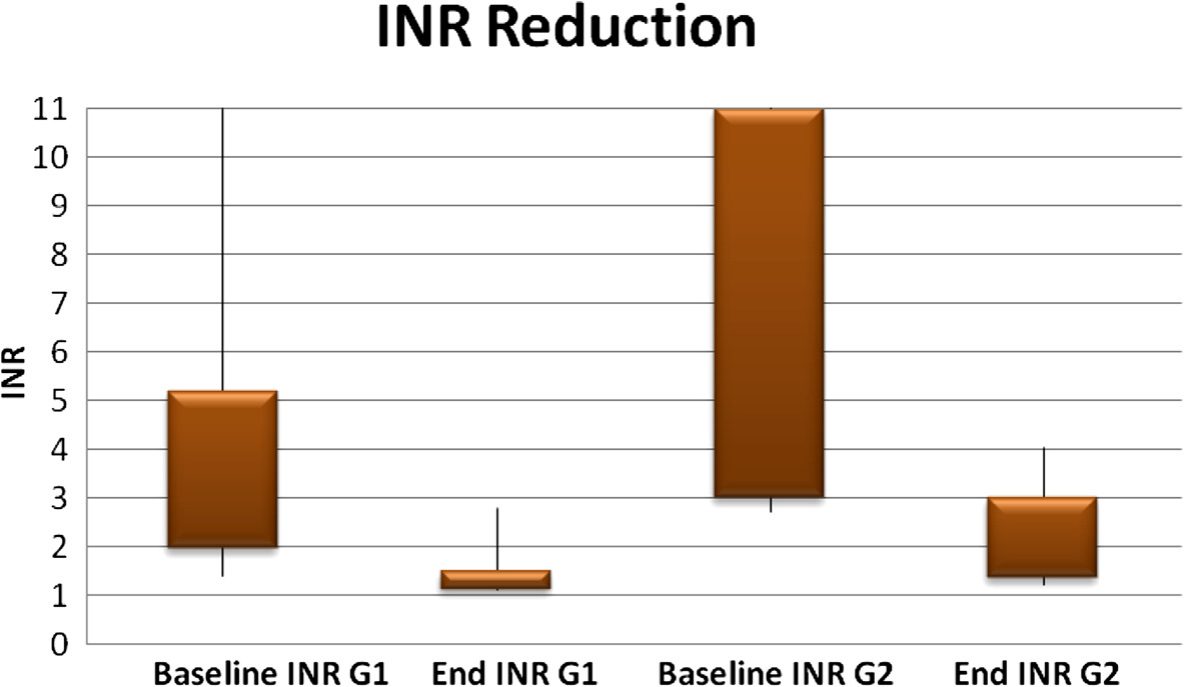
**INR reduction in two target groups (G1 = target INR ≤ 1.5, G2 = target INR 1.6**-**2.1). **

It appeared that patients in group 1 had a significantly lower baseline INR than patients in group 2. Group 2 patients, on the other hand, had a baseline INR > 7.5 in 53% of the cases (Figure 1). This supra-therapeutic range of INR may have contributed to the different kind of bleeding witnessed in this patient group.

In our cohort, the achieved INR values were outside the desired range of 1.6-2.1 for most patients in group 2 who were treated with Co-fact^©^. Patients in group 2 by definition did not have life-threatening bleeding or bleeding requiring immediate intervention. For these patients, we propose a more conservative approach. For instance, after administration of a fixed bolus dose, a new blood sample for INR assessment can be drawn. When the new INR value is known (which usually occurs within 30 min, but can be even quicker when using point-of-care measurements in the ED), patients requiring extra VKA reversal after the first bolus can be treated accordingly. With an initially lower amount of Co-fact^©^ administered, the percentage of over-treated patients can be reduced. The total delay with this method is not more than 30 min, and overdosing of Co-fact^©^ can be reduced, which could reduce complications of overdosing and is cost effective.

Based on the number of patients in our cohort, it is difficult to draw very solid conclusions. Also, the INR target groups were not the same size, which makes comparison between these groups less powerful.

However, this prospective cohort study was intended to test our hypothesis and give possible directions for future research. The results support the idea that treatment of patients on vitamin K antagonists with Co-fact^©^ could benefit from a slightly different approach, as suggested above.

## Competing interests

The authors declare that they have no competing interests.

## Authors’ contributions

FR and JM participated in setting up the study design. FR gathered and analysed the data. All authors participated in writing the article. All authors read and approved the final manuscript.

## Supplementary Material

Additional file 1 **Appendix. **Manufacturer dosing regimen of Co-fact^©^. Click here for file
