# Supplementary material for: INR reduction after prothrombin complex concentrate (Co-fact©) administration: comparison of INR outcomes in different patient categories at the emergency department
Source: Int J Emerg Med. 2013 May 10;6:14. doi: 10.1186/1865-1380-6-14 (PMC3655866; doi:10.1186/1865-1380-6-14)
Supplement: Additional file 1 — Appendix. Manufacturer dosing regimen of Co-fact©. [file 1865-1380-6-14-S1.docx]

**Additional file 1: Manufacturer dosing regimen of Co-fact^©^**

| Recommended dose of Co-fact ® in ml to achieve target INR ≤ 2.1 | | | | | | | | | | | | | |
| --- | --- | --- | --- | --- | --- | --- | --- | --- | --- | --- | --- | --- | --- |
| Baseline  INR  Weight | 7.5 | 5.9 | 4.8 | 4.2 | 3.6 | 3.3 | 3.0 | 2.8 | 2.6 | 2.5 | 2.3 | 2.2 |  |
| 50 kg | 40 | 40 | 40 | 30 | 30 | 30 | 20 | 20 | - | - | - | - |  |
| 60 kg | 50 | 50 | 40 | 40 | 30 | 30 | 30 | 20 | - | - | - | - |  |
| 70 kg | 60 | 50 | 50 | 50 | 40 | 40 | 30 | 30 | - | - | - | - |  |
| 80 kg | 60 | 60 | 60 | 50 | 50 | 40 | 40 | 30 | - | - | - | - |  |
| 90 kg | 60 | 60 | 60 | 60 | 50 | 50 | 40 | 30 | - | - | - | - |  |
| 100 kg | 60 | 60 | 60 | 60 | 60 | 50 | 40 | 40 | - | - | - | - |  |

| Recommended dose of Co-fact ® in ml to achieve target INR ≤ 1.5 | | | | | | | | | | | | |
| --- | --- | --- | --- | --- | --- | --- | --- | --- | --- | --- | --- | --- |
| Baseline  INR  Weight | 7.5 | 5.9 | 4.8 | 4.2 | 3.6 | 3.3 | 3.0 | 2.8 | 2.6 | 2.5 | 2.3 | 2.2 |
| 50 kg | 60 | 60 | 60 | 50 | 50 | 50 | 40 | 40 | 30 | 30 | 30 | 30 |
| 60 kg | 80 | 70 | 70 | 60 | 60 | 60 | 50 | 50 | 40 | 40 | 40 | 30 |
| 70 kg | 90 | 80 | 80 | 70 | 70 | 70 | 60 | 60 | 50 | 40 | 40 | 40 |
| 80 kg | 100 | 100 | 90 | 90 | 90 | 80 | 80 | 70 | 60 | 50 | 50 | 40 |
| 90 kg | 100 | 100 | 100 | 90 | 90 | 90 | 80 | 80 | 70 | 60 | 50 | 40 |
| 100 kg | 100 | 100 | 100 | 100 | 100 | 90 | 90 | 80 | 70 | 70 | 60 | 50 |
